# Addressing the pervasive scarcity of structural annotation in eukaryotic algae

**DOI:** 10.1038/s41598-023-27881-0

**Published:** 2023-01-30

**Authors:** Taehyung Kwon, Erik R. Hanschen, Blake T. Hovde

**Affiliations:** Genomics and Bioanalytics, Bioscience Division, Los Alamos National Laboratory, Los Alamos, NM USA

**Keywords:** Bioinformatics, Comparative genomics, Phylogenetics, Genetic databases

## Abstract

Despite a continuous increase in algal genome sequencing, structural annotations of most algal genome assemblies remain unavailable. This pervasive scarcity of genome annotation has restricted rigorous investigation of these genomic resources and may have precipitated misleading biological interpretations. However, the annotation process for eukaryotic algal species is often challenging as genomic resources and transcriptomic evidence are not always available. To address this challenge, we benchmark the cutting-edge gene prediction methods that can be generalized for a broad range of non-model eukaryotes. Using the most accurate methods selected based on high-quality algal genomes, we predict structural annotations for 135 unannotated algal genomes. Using previously available genomic data pooled together with new data obtained in this study, we identified the core orthologous genes and the multi-gene phylogeny of eukaryotic algae, including of previously unexplored algal species. This study not only provides a benchmark for the use of structural annotation methods on a variety of non-model eukaryotes, but also compensates for missing data in the current spectrum of algal genomic resources. These results bring us one step closer to the full potential of eukaryotic algal genomics.

## Introduction

The term “eukaryotic algae” defines a polyphyletic group of photoautotrophic organisms that stem from an event of primary cyanobacterial endosymbiosis^1^, followed by a rich history of secondary and tertiary endosymbiotic events. In this study, we use the term to encompass diverse forms of organisms that originated from the deepest branches of the eukaryotic tree of life. The primary endosymbiosis event formed the Archaeplastida lineage, currently composed of Viridiplantae, Rhodophyta, and Glaucophyta^2,3^. Afterwards, red algae of the Archaeplastida are hypothesized to have been engulfed by the “Chromalveolata”^4^, the ancestors of red alga-derived secondary plastids. This eukaryote-eukaryote endosymbiosis event gave birth to the current Haptophyta, Cryptophyta, and SAR clade (Stramenopiles, Alveolata, Rhizaria)^2–4^. Since the photoautotrophic capability of the primary plastids was spread to the eukaryotic algal lineages through serial endosymbiotic gene transfers (EGT), the present-day eukaryotic algal lineages share a long history of adaptation to the photoautotrophy^2,5^. Alongside this core algal biology, deep branch lengths of the algal species are enriched with genome mutation, gene duplication/loss, EGT, and horizontal gene transfer events (HGT) that vary between lineages. This has contributed to the enormous genotypic and phenotypic biodiversity of the eukaryotic algae^2,5^, which has brought versatility in adaptation to various environments.

With the enormous diversity and underlying value of eukaryotic algae, growing attention and recent innovations in sequencing technology have allowed algal genomics to prosper, with numerous algal genome assemblies becoming accessible. Nevertheless, a recent study reported that most of the currently available algal genomes remain without annotations^6^ as the annotation process of an algal genome is often challenging. Without structural annotation of coding regions, eukaryotic genomes are prone to the false discoveries of genetic information due to the complex exon–intron nature. Therefore, these unannotated genomes likely remain untouched in terms of functional, comparative, or evolutionary analyses. In addition, the quantity and quality of these genomic resources are far from evenly distributed across taxa^6^. This scarcity and uneven data distribution has obscured the full potential of the current algal genome diversity and limited inter-species comparative analysis^7,8^. Moreover, this challenge has impeded the application of state-of-the-art bioinformatics methods that are actively used in other taxonomic realms. In this sense, we believe that one of the most urgent goals in algal genomics is to provide structural annotation for unannotated genome assemblies.

Computational methods for eukaryotic genome annotation have been developed for decades to accurately resolve exon–intron boundaries. These methods, including well-known ones such as AUGUSTUS^9^, Maker2^10^, Braker2^11^, and GeneMark series^12–14^, perform gene finding based on a glimpse of coding region structure, referred to as “hints”. Different pipelines are recommended according to the type of hint data. For example, AUGUSTUS and GeneMark-ES use intrinsic hints detected from sheer genome sequence itself (ab initio)^9,14^, while Braker2 and Maker2 allow comprehensive pipelines using transcriptome or proteome-based hints^10,11^. However, most of the benchmarks of these tools have focused on performances in model organisms^15,16^.

Throughout this study, we assess the performances of cutting-edge annotation tools when training eukaryotic algal gene models without the requirement of species-specific extrinsic evidence. We test two annotation tools, AUGUSTUS and Braker2, with a promising quality assessment tool for assembly/annotation, Benchmarking Universal Single-Copy Orthologs (BUSCO)^17^. AUGUSTUS, known for superior performance in ab initio eukaryotic gene prediction, is capable of gene model training with intrinsic hints detected from BUSCO analysis^9,18^. Braker2, one of the most comprehensive annotation tools, implements different modes of annotation such as modes using protein hints and/or transcriptomic hints, or ab initio mode. Our approach focuses on an annotation process and its benchmark that targets a variety of eukaryotic algal species, is generalizable, and does not necessitate species-specific evidence. Thus, this study will be advantageous to the understudied algal clades that have limited genomic and transcriptomic resources available.

In this study, we attempt to retrieve the pervasive scarcity of structural annotation in the eukaryotic algae. The main objectives of this study are (1) to assess the performance of the annotation methods in high-quality eukaryotic algal genomes; (2) to recover missing structural annotations in 135 algal genome assemblies; and (3) to establish the eukaryotic algal orthology and phylogeny using these newly predicted algal genes. While our finding provides a benchmark for the application of cutting-edge annotation methods to various non-model eukaryotes, it also performs a heavyweight task for the algal research community by providing valuable but currently missing data. We believe that this study will nourish numerous downstream applications comprising comparative, functional, and evolutionary genomics.

## Results

### Summary of the eukaryotic algal genome database

**Figure 1:**
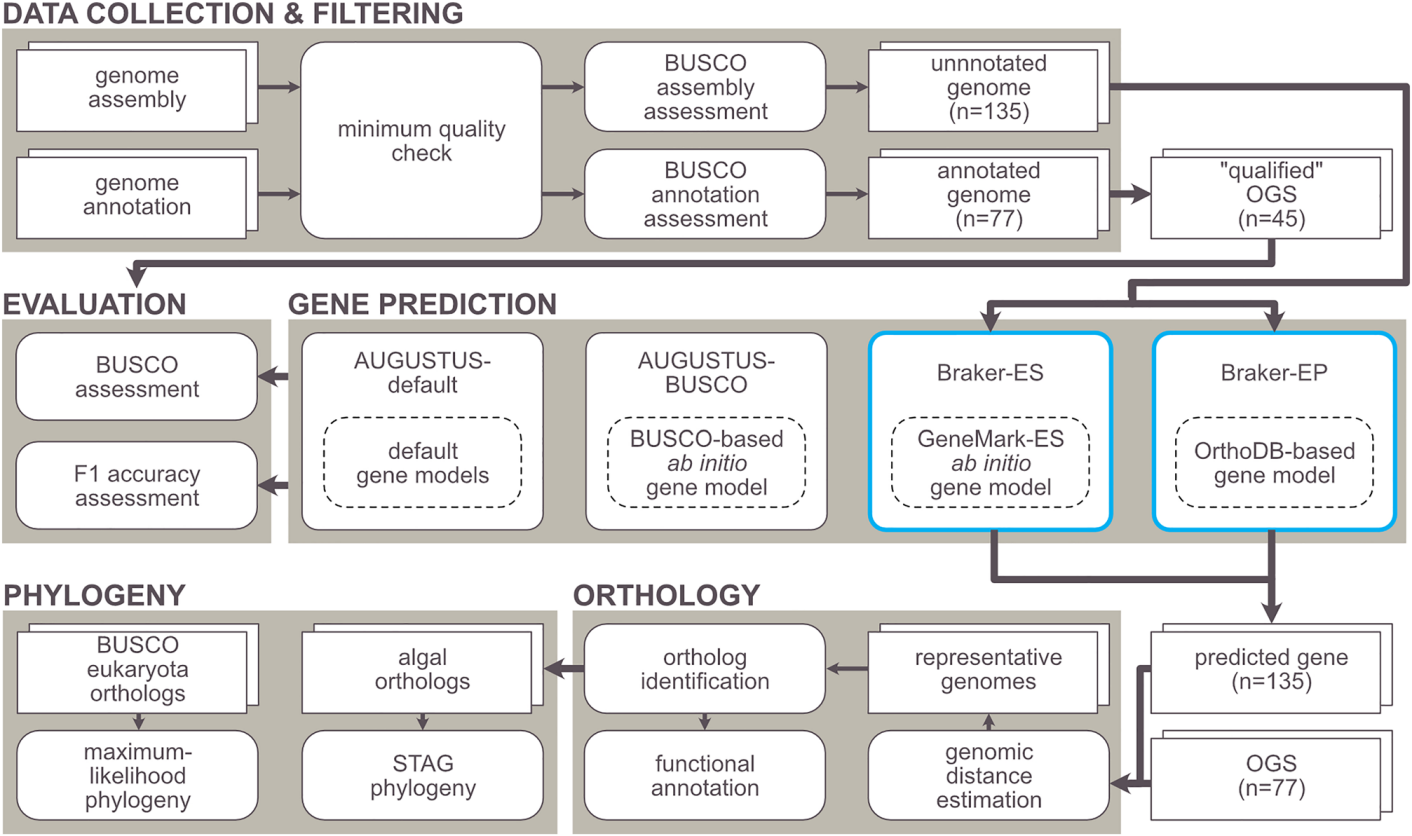
Flowchart of the present study. OGS denotes official gene set.

We collected 257 eukaryotic algal genome assemblies from public databases including the NCBI RefSeq/GenBank database^19,20^ (see *Database* column of Supplementary Tables S1 and S2). Available genome annotations and protein sequences that correspond to the genome assemblies were also retrieved from the databases. Before performing any analyses on these genomic resources, we excluded low-quality genome assemblies via minimum quality check and BUSCO genome mode (Fig. 1).

For the minimum quality check, we first excluded 29 incomplete NCBI assemblies: six partial assemblies, nine assemblies derived from metagenome sources, 12 assemblies derived from single-cell sources, and two assemblies derived from environmental samples (see *Assembly assessment* column of Supplementary Table S2). Next, we excluded five assemblies with contig N50 values smaller than the median gene length of eukaryotic algae, as suggested by Yandell et al.^21^ (see *Assembly assessment* column of Supplementary Table S2). The median gene length of 1.608 Kb was calculated from genome annotations of 18 genome assemblies with contig N50 of 100 Kb or more. As a result, 223 genome assemblies, 210 of which are soft-masked, passed the minimum quality check.

**Figure 2:**
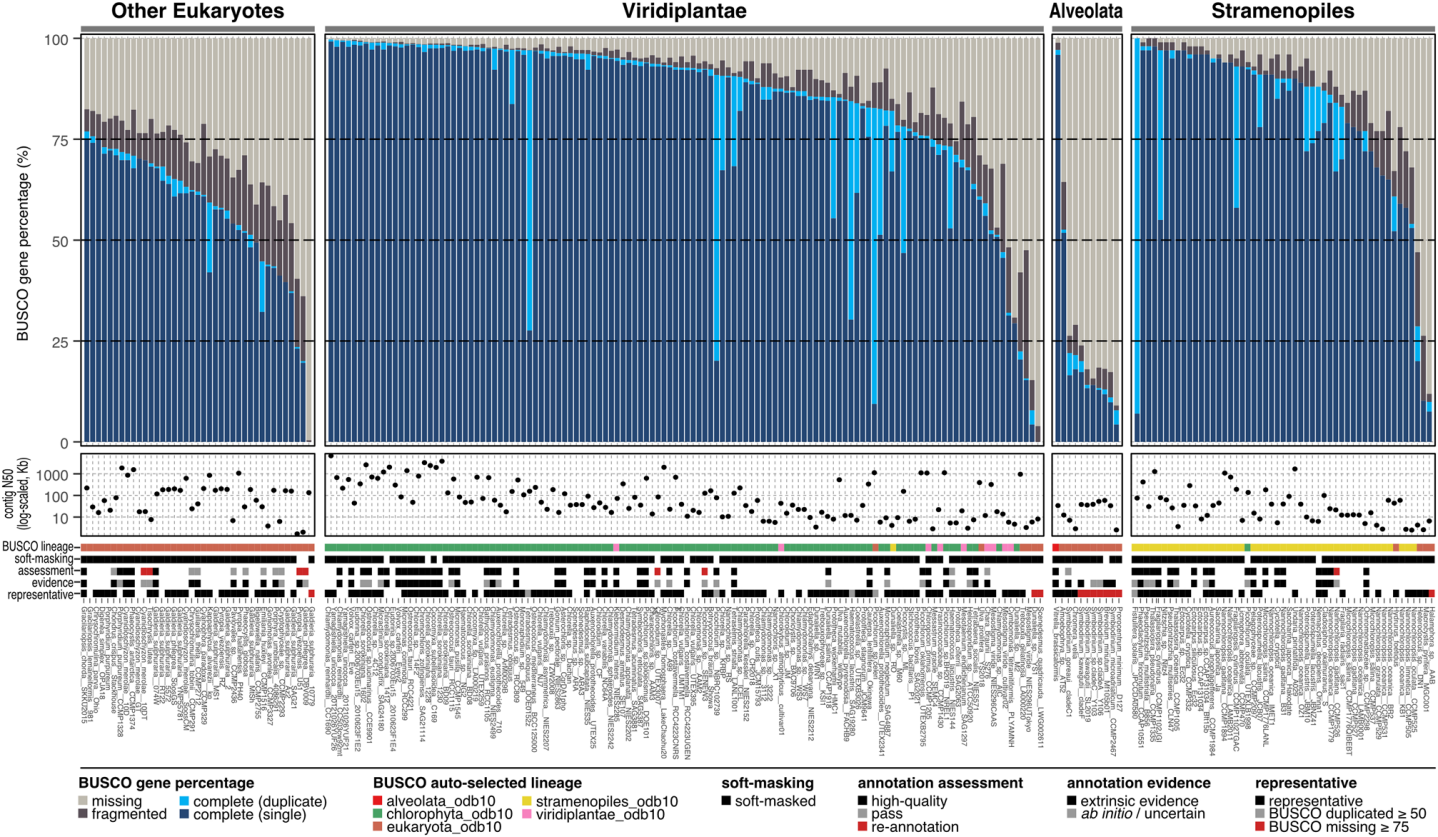
BUSCO assessment results of genome assemblies. The top panel displays BUSCO genome assessment with the automatically selected BUSCO lineages. The second panel displays log-scaled contig N50 values. Each row of the third panel illustrates information described in Supplementary Table S1. BUSCO lineage row displays automatically selected BUSCO lineages during BUSCO genome assessment. Soft-masking row displays whether the genome is soft-masked or not. Assessment row displays BUSCO protein assessment result of the structural annotation. Evidence row displays evidence used for annotation of the official gene set. Representative row indicates whether the genome is representative genome among closely related genomes.

For 83 genome assemblies with structural annotations, we examined (1) anomalies in core annotation features such as coding sequence, exon, and transcript and (2) correspondences between genome assembly, genome annotation, and protein sequences. As a result, we excluded five annotations, leaving 78 annotations (see *Annotation assessment* column of Supplementary Table S1). We note that annotations excluded during any quality assessments in this study were later generated by the annotation process of this study (denoted as “reannotation” in *Assembly type* column of Supplementary Table S1 and Fig. 2). We also examined the engagement of extrinsic evidence (e.g., RNA-seq) in the original genome annotation processes of the annotated genomes (see *Annotation evidence* column of Supplementary Table S1 and Fig. 2).

According to the NCBI taxonomy, we classified our dataset hierarchically by taxonomic supergroups, groups, and subgroups (Supplementary Table S1). The Viridiplantae supergroup is composed of the Chlorophyta and Streptophyta groups. The Stramenopiles supergroup is solely composed of the Ochrophyta group. The Alveolata supergroup/group is composed of Dinophyceae and Colpodellida subgroups. The Other eukaryotes supergroup contains eukaryotic algal species that are placed outside of the other supergroups defined above, which comprises five taxonomic groups: Rhodophyta, Haptophyta, Cryptophyta, Rhizaria, and Glaucophyta.

### BUSCO assessment of genome assemblies

In addition to the minimum quality check, we performed BUSCO analyses to assure the quality of the dataset for downstream analysis (Fig. 1). Genome assemblies and annotations excluded in the following section are also noted in Supplementary Tables S1 and S2, respectively. We used automatic BUSCO lineage selection to perform subsequent BUSCO analyses, results of which are also summarized in the *BUSCO auto-selected lineage*, *BUSCO complete (duplicate)*, and *BUSCO missing* columns of Supplementary Table S1.

For all genome assemblies, we performed BUSCO genome assessment, which quantifies orthologous contents only within a genome sequence (Fig. 2). A total of five OrthoDB v10 datasets related to algal species were assigned by BUSCO (Fig. 2): eukaryota_odb10 (the number of genes = 255), viridiplantae_odb10 (n = 425), chlorophyta_odb10 (n = 1519), alveolata_odb10 (n = 171), and stramenopiles_odb10 (n = 100). We note that the eukaryota_odb10 dataset was manually assigned to *Galdieria sulphuraria* 10779 and *Scenedesmus quadricauda* LWG002611, as not enough eukaryota_odb10 complete genes were found in these assemblies to determine lineages. In the Viridiplantae and Stramenopiles supergroups, genome assemblies with BUSCO missing rates lower than 75% were mainly assigned with OrthoDB datasets corresponding to their supergroups (Fig. 2): Viridiplantae (chlorophyta_odb10, n = 106; viridiplantae_odb10, n = 9; eukaryota_odb10, n = 4; stramenopiles_odb10, n = 1), Stramenopiles (chlorophyta_odb10, n = 1; eukaryota_odb10, n = 3; stramenopiles_odb10, n = 46), Alveolata (eukaryota_odb10, n = 3; alveolata_odb10, n = 1). However, genome assemblies with BUSCO missing rates over 75% were all analyzed with eukaryota_odb10 (Fig. 2): Viridiplantae (n = 2), Stramenopiles (n = 1), Alveolata (n = 7). Therefore, we filtered out 11 genome assemblies with BUSCO missing rates over 75% from downstream analyses (Supplementary Table S2), which resulted in a final set of 212 genome assemblies.

On average, the results of BUSCO genome assessment using corresponding OrthoDB datasets (Fig. 2) showed lower missing rates compared to those using eukaryota_odb10 (Supplementary Fig. S1), which supports the merit of the use of corresponding OrthoDB datasets. In the BUSCO analyses with corresponding OrthoDB datasets, the mean BUSCO missing rates were 31.31%, 10.77%, 69.18%, and 12.66% in the Other Eukaryotes, Viridiplantae, Alveolata, and Stramenopiles supergroups, respectively (Fig. 2). The mean BUSCO missing rates with eukaryota_odb10 for all supergroups were higher than those using the taxonomically corresponding BUSCO datasets: 19.16%, 71.54%, and 26.36% in Viridiplantae, Alveolata, and Stramenopiles supergroups, respectively (Supplementary Fig. S1). The Alveolata supergroup had the highest mean BUSCO missing rate among all supergroups. For the genomes of genus *Symbiodinium,* we observed BUSCO missing rates over 50% of regardless of the genome continuity (Fig. 2), indicating a poor representation of *Symbiodinium* genes in the eukaryota_odb10.

### BUSCO assessment of genome annotations

**Figure 3:**
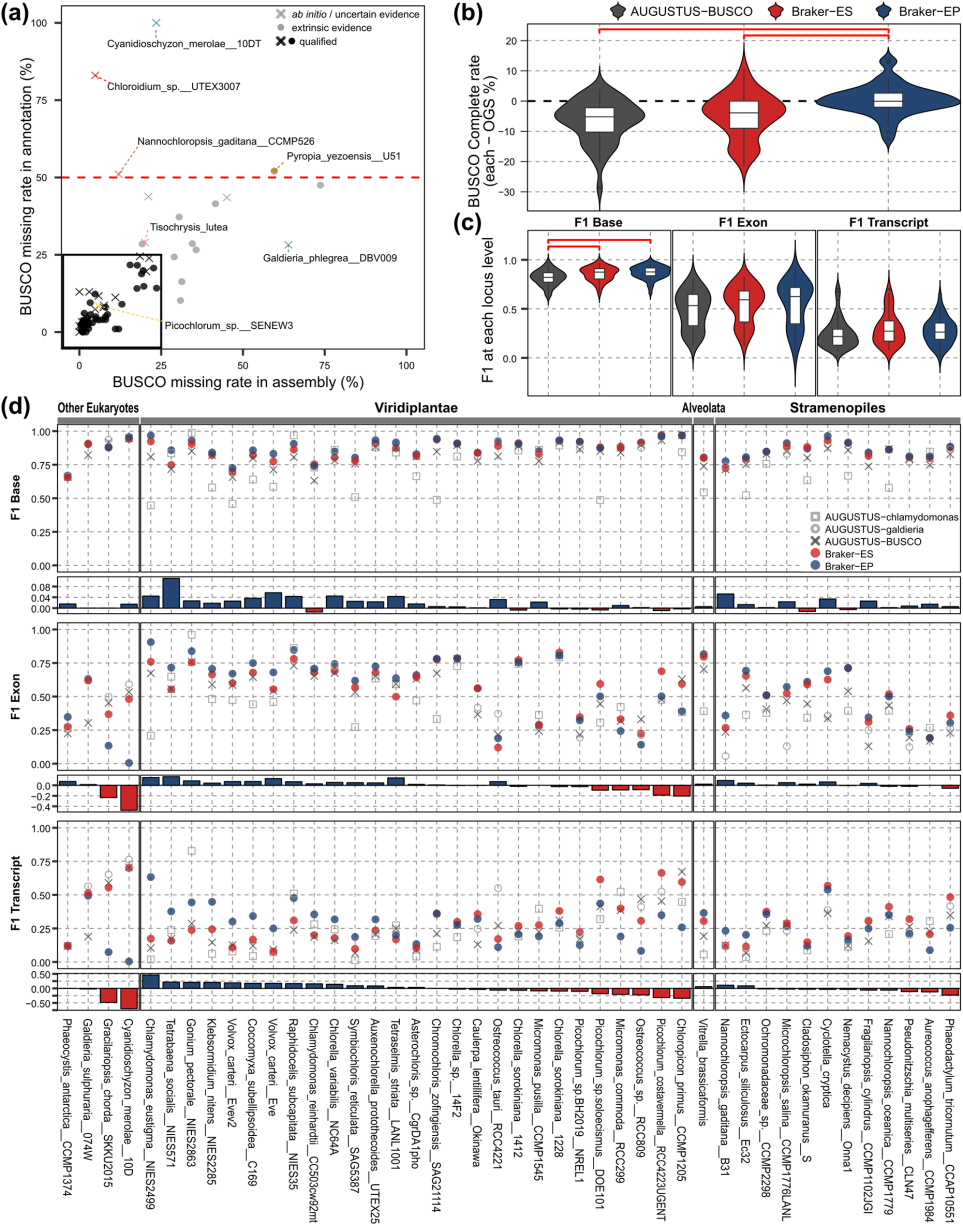
Evaluation of different gene prediction methods. (**a**) Missing rates from the BUSCO genome assessment (x-axis) and protein assessment (y-axis) in 83 annotated genomes. Genomes located in the black rectangle are considered high-quality official gene sets. High-quality official gene sets annotated using extrinsic evidence were marked as “qualified” official gene sets that are further used for the evaluation. Genomes indicated with their identifiers are re-annotated, of which details are summarized in *Annotation assessment* of Supplementary Table S1. (**b**) BUSCO complete rate comparison between the official gene set and three newly predicted gene sets. Positive values indicate that the newly predicted gene set outperformed the official gene set. Red brackets indicate significant difference (Wilcoxon rank-sum test *p*-value < 0.05). (**c**) F1 score comparison at all loci levels between three newly predicted gene sets. Red brackets indicate significant difference (Wilcoxon rank-sum test *p*-value < 0.05). (**d**) F1 scores of predicted gene sets at all loci levels. Only the best performing AUGUSTUS-default gene models are displayed. Bar graphs at the bottom of each F1 score plot display differences in F1 between Braker-EP and Braker-ES. Positive values indicate that Braker-EP gene model outperforms Braker-ES gene model.

For the annotated assemblies, we performed BUSCO protein assessment which quantifies orthologous contents from a set of protein sequences extracted based on structural annotations (Fig. 3a). Here, we used OrthoDB datasets automatically selected during the BUSCO genome assessment (Fig. 2). Based on this result, we selected high-quality structural annotation and excluded low-quality structural annotations from downstream analysis (see *Annotation assessment* column of Supplementary Table S1). Genomes with low missing rates from BUSCO genome assessment and high missing rates from BUSCO protein assessment (shown in the upper left of the coordinate planes in Fig. 3a) may result from incomplete structural annotations. The opposite cases (shown in the lower right of the coordinate planes in Fig. 3a) indicate that BUSCO genome assessment could not detect all genes possibly due to the complexity of coding sequence structures.

A total of four genome assemblies had BUSCO protein missing rates over 50% (Fig. 3a). Two of them also failed the minimum quality check due to missing core annotation features: *Cyanidioschyzon merolae* 10DT and *Chloroidium* sp. UTEX3007. One of the other two structural annotations with high BUSCO missing rates, *Nannochloropsis gaditana* CCMP526, had higher BUSCO protein missing rates (51.0%) than its BUSCO genome missing rates (12.0%) (Fig. 3a). However, *Pyropia yezoensis* U51 had high missing rates in both genome (59.6%) and protein (52.1%), which may have resulted from the low assembly quality (Fig. 3a). Therefore, we did not reannotate structural annotation of *Pyropia yezoensis* U51 in downstream analyses. In total, six structural annotations were replaced with the newly predicted gene sets as they failed the minimum quality check and/or BUSCO protein assessment (denoted as “reannotation” in *Assembly type* column of Supplementary Table S1). After performing the minimum quality check and BUSCO assessments, 212 genome assemblies and 77 structural annotations were selected for downstream analyses.

### Evaluation of species-specific gene prediction methods

A total of 135 genome assemblies remained (1) without official gene sets (OGS) (n = 129), or (2) with OGSs that are excluded during filtering (n = 6), which in total takes account for 63.68% of the eukaryotic algal genome dataset. In addition, 21 of 31 algal taxonomic subgroups in our algal genome database are “data-sparse” subgroups, referring to the subgroups with three or fewer genome assemblies available. These data-sparse subgroups were composed of 29 genome assemblies, and 16 of them were without structural annotation.

To resolve this pervasive lack of structural annotations, we evaluated three generalizable methods to predict genes in various eukaryotic algal species: (1) AUGUSTUS gene prediction with a BUSCO-based predicted gene model (AUGUSTUS-BUSCO), (2) Braker2 gene prediction with ab initio gene finding algorithm of GeneMark-ES (Braker-ES), and (3) Braker2 gene prediction with protein extrinsic evidence from OrthoDB v10 (Braker-EP) (Fig. 1). AUGUSTUS-BUSCO includes a preceding BUSCO genome mode analysis to train species-specific gene model for gene prediction. Braker-EP includes a parsing process of the OrthoDB v10 genes to generate clade-specific protein hints, while Braker-ES finds trainable hints within the genome assembly itself^14^. These methods can universally predict genes from any eukaryote algal species without the requirement of species-specific data, which is particularly useful for structural annotation of data-sparse species. To compare with these methods, we also generated gene models using three default AUGUSTUS gene models: AUGUSTUS-chlamydomonas, AUGUSTUS-galdieria, and AUGUSTUS-tetrahymena.

All gene models were generated from 45 high-quality genomes and compared with the OGSs of these genomes, namely “qualified” genomes (see *Assembly type* column of Supplementary Table S1) (Fig. 1). These “qualified” genomes showed BUSCO missing rates less than 25% in both BUSCO genome and protein assessments (Fig. 3a) and were reported to be annotated using extrinsic evidence (see *Annotation evidence* column of Supplementary Table S1 and Fig. 2). First, we compared BUSCO protein assessment results by subtracting BUSCO complete rates of the OGSs from those of the predicted gene sets (Fig. 3b). On average, BUSCO complete rates of Braker-EP gene sets were performed better than those of OGSs: mean difference = 0.41%, standard deviation (SD) = 4.99%. Compared to AUGUSTUS-BUSCO and Braker-ES, Braker-EP gene sets showed significantly higher BUSCO complete rates than both Braker-ES (− 4.68 ± 7.37%, Wilcoxon rank-sum test *p*-value = 5.19e^−4^) and AUGUSTUS-BUSCO gene sets (− 6.48 ± 6.98%, *p*-value = 1.42e^−6^). Braker-EP also showed the lowest average BUSCO missing rates among the three predicted gene sets (Supplementary Fig. S2). Therefore, in regard to the core orthologous genes, Braker-EP gene prediction outperformed the other two methods and was comparable to the OGS.

To assess accuracy using the entire set of the predicted genes, we compared all predicted genes against all OGS genes at three different levels of loci (Figs. 3c and d). In the accuracy measurement, a true positive match between a predicted gene and an OGS gene refers to a significant overlap at the loci level (base, exon, or transcript), details of which are described in previous studies^22,23^. To summarize the accuracy evaluation, we used the “F1 score”^24^, the harmonic mean of “sensitivity” (the ratio of true positive matches to the OGS) and “precision” (the ratio of true positive matches to the newly predicted genes). It should be noted that this evaluation process is subject to the quality of the OGS, as we assume that the OGS is ground truth.

On average, F1s of AUGUSTUS-BUSCO gene models were lower than those of Braker-EP and Braker-ES gene models, while F1s of Braker-ES and Braker-EP gene models did not significantly differ (Table 1 and Fig. 3c). Particularly, F1s at base loci level of AUGUSTUS-BUSCO were significantly lower than those of both Braker-EP and Braker-ES (Table 1 and Fig. 3c), which supports that both Braker-based methods outperform AUGUSTUS-BUSCO.

**Table 1:** Accuracy of the gene prediction methods at different loci levels.

Statistics	Braker-EP (mean ± SD)	Braker-ES	AUGUSTUS-BUSCO
F1-base	0.87 ± 0.07	0.85 ± 0.07	0.82 ± 0.08
F1-exon)	0.55 ± 0.23	0.55 ± 0.19	0.49 ± 0.19
F1-transcript	0.27 ± 0.14	0.3 ± 0.17	0.25 ± 0.15

Although Braker-EP gene models showed higher F1 than Braker-ES gene models at base level (Fig. 3c), Braker-EP gene models of a few genomes showed low F1s at exon and transcript levels compared to the gene models using other methods (Fig. 3d): *Cyanidioschyzon merolae* 10D*, Gracilariopsis chorda* SKKU2015, *Chloropicon primus* CCMP1205, *Picochlorum costavermella* RCC4223UGENT, *Ostreococcus* sp. RCC809, and *Phaeodactylum tricornutum* CCAP10551. We observed that Braker-EP gene models of these genomes predicted an excessive number of introns compared to the actual number of introns in their OGSs. For example, the OGS of *Cyanidioschyzon merolae* 10D contained only 24 introns across 4292 genes, whereas the Braker-EP predicted gene set contained 5203 introns across 3970 genes, which resulted in 99.6% of the predicted introns being false positives. In a similar fashion, more than 40% of predicted introns were false positives in Braker-EP gene models of these five genomes: 84.3% (*Gracilariopsis chorda* SKKU2015), 78.1% (*Ostreococcus* sp. RCC809), 58.3% (*Chloropicon primus* CCMP1205), 52.0% (*Phaeodactylum tricornutum* CCAP10551), and 43.2% (*Picochlorum costavermella* RCC4223UGENT). This result was in contrast with the false positive introns in the genomes of which Braker-EP gene models outperformed the others: *Chlamydomonas eustigma* NIES2499 (2.2%) and *Tetrabaena socialis* NIES571 (11.2%). Poor performance of Braker-EP would have stemmed from misleading protein hints, potentially caused by difficult spliced alignments^25^. F1 accuracy of Braker-EP and Braker-ES showed correlations with genome size. Both Braker-EP and Braker-ES showed significant negative correlations with genome size at the base level (Supplementary Fig. S3): Braker-EP, Pearson’s correlation coefficient *r* = − 0.35, *p*-value = 0.022; Braker-ES, *r* = − 0.38, *p*-value = 0.011. Interestingly, F1 of Braker-ES showed a significant negative correlation with genome size at the transcript level (*r* = − 0.40, *p*-value = 0.0077) (Supplementary Fig. S3). On the other hand, F1 of Braker-EP showed almost zero correlation with genome size at the transcript level (*r* = − 0.017, *p*-value = 0.91) (Supplementary Fig. S3). This result suggests that Braker-ES gene models performed well with small genomes. F1s of Braker-EP or Braker-ES were not significantly correlated with genome continuity or BUSCO complete rates, which supports the robustness of these Braker-based methods (Supplementary Fig. S3).

We showed that Braker-EP underperformed all other methods for the genomes with a small number of introns, possibly due to misleading protein spliced alignment of the OrthoDB v10 protein hints. For these genomes, Braker-ES performed well as an alternative to Braker-EP (Fig. 3d). In this sense, the BUSCO complete rate of a genome assembly can work as an indicator of the quantity of complete OrthoDB genes within the genome and protein hints. Although not significant, we observed an opposing trend of correlation between Braker-EP and Braker-ES; BUSCO complete rates showed a negative correlation with F1s of Braker-ES gene models at the transcript level (*r* = − 0.29, *p*-value = 0.060), while those of Braker-EP gene models showed a weak positive correlation with BUSCO complete rates (*r* = 0.12, *p*-value = 0.45) (Supplementary Fig. S3). Four of the six genomes showing severely low Braker-EP F1 also showed low BUSCO complete rates: *Cyanidioschyzon merolae* 10D (71.4%)*, Gracilariopsis chorda* SKKU2015 (76.9%), and *Chloropicon primus* CCMP1205 (75.8%) (Figs. 2 and 3d). The weak correlation between F1 and BUSCO complete rate suggests that not all Braker-EP gene models with low F1 showed low BUSCO complete rates: *Picochlorum costavermella* RCC4223UGENT (92.7%), *Ostreococcus* sp. RCC809 (97.1%), and *Phaeodactylum tricornutum* CCAP10551 (98.0%). Therefore, we devised a simple strategy to mix Braker-EP and Braker-ES based on BUSCO complete rate of genome assembly. With different BUSCO complete rate cutoffs ranging from 60 to 100%, we used Braker-ES gene model for the genomes with BUSCO complete rate smaller than the cutoff and used Braker-EP gene model for the genomes with BUSCO complete rate of the cutoff or larger (Supplementary Fig. S4). Based on this result, we selected a strategy with BUSCO complete rate cutoff of 90% where we use Braker-ES for genomes with BUSCO complete rate < 90% and Braker-EP for genomes with BUSCO complete rate ≥ 90%. Since the accuracy evaluation using the entire gene set cannot directly compare the gene model with the OGS, we could not conclude whether the Braker-based gene models outperform the OGSs. Nevertheless, the strategy to mix two Braker-based methods offers a robust, reproducible, and generalized solution for automated gene prediction in non-model eukaryotic algae.

### The eukaryotic algal orthology

For orthology and phylogeny analyses of eukaryotic algal species, we selected 149 representative algal genomes to mitigate phylogenetic unevenness in our dataset. We first estimated pair-wise genetic distance between genomes using MASH^26^. We then clustered genomes with 96% or more pair-wise identities and selected a representative genome with the highest BUSCO genome complete rate within each cluster, which is a method previously used in OrthoDB sample selection^27^. After excluding highly missing (BUSCO missing rate over 75%) or highly duplicated (BUSCO complete duplicated rate over 50%) genomes, we selected one representative genome with the best BUSCO complete rates within each cluster.

**Figure 4:**
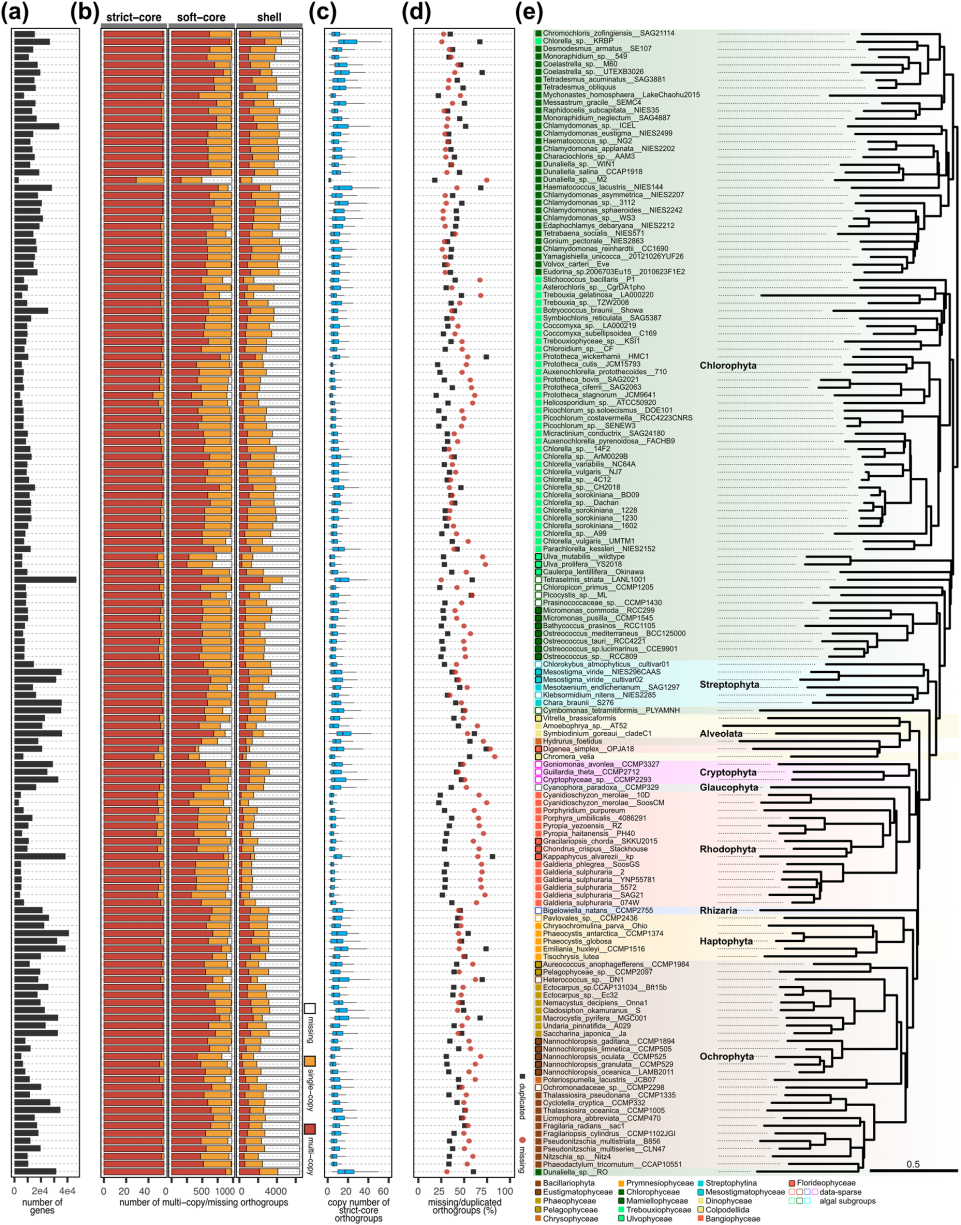
Per-species orthology statistics with STAG phylogenetic tree. (**a**) The number of genes. (**b**) The number of multi-copy, single-copy, and missing OGs across the strict-core, soft-core, and shell OG clusters. (**c**) Boxplot of copy numbers of the strict-core OGs. Outliers were omitted. (**d**) Duplication rates (black) and missing rates (red) calculated in the strict-core, soft-core, and shell OG clusters. Missing rates were calculated with all OGs (n = 7432). Duplication rates were calculated only within the OGs found in each genome. (**e**) STAG tree reconstructed using 54 strict-core OGs. Taxonomic groups are indicated by shaded rectangles. Taxonomic subgroups are indicated by a colored square.

As a result, we explored the algal orthology using 149 gene sets: 58 OGSs and 91 newly predicted gene sets using two Braker-based methods. In this analysis, we employed a concept of orthogroup (OG), representing a group of orthologous genes (hereafter, orthologs) and encompassing from one-to-one orthologs to many-to-many orthologs. OGs were sorted into four clusters based on the number of species sharing the OG, as defined in a previous study^28^: “strict-core”, “soft-core”, “shell”, and “cloud”. The strict-core OGs represent a set of conserved orthologs across all 149 representative genomes. The soft-core OGs represent a set of conserved orthologs across at least 95% of the representative genomes (shared by from 142 to 148 genomes). The shell OGs contain a broad range of conservation from 15% to less than 95% of the members of the eukaryotic algae (shared by from 23 to 141 genomes). Lastly, the cloud OGs contain mostly lineage-specific orthologs that are detected in less than 15% of the genomes. A total of 2,061,744 of 2,288,060 genes (90.10%) were assigned to 116,433 OGs, mainly to the cloud cluster (93.62%, n = 109,001) (Supplementary Table S3). The other OGs were assigned to the shell cluster (n = 6876, 5.91%), soft-core cluster (n = 502, 0.43%), and strict-core cluster (n = 54, 0.046%) (Supplementary Fig. S5 and Table S3). Among 109,001 cloud OGs, 46,824 OGs were species-specific, referring to an OG found in only one genome (Supplementary Table S3). The number of genes varied across genomes from 3252 to 46,705, which has a large spread from the quality of genome data and diversity of intrinsic genomic nature (Fig. 4a). Genome size was correlated with the number of genes (Pearson’s correlation coefficient *r* = 0.60) (Supplementary Fig. S6). The number of genes showed positive correlations with the number of OGs (*r* = 0.83) (Supplementary Fig. S6). The percentages of the cloud OG and species-specific OG were positively correlated with the number of genes (*r* = 0.71 and 0.69, respectively), which contrasts the percentages of the strict-core, soft-core, and shell OGs being negatively correlated with the number of genes (*r* = − 0.72, − 0.77, and − 0.61, respectively) (Supplementary Fig. S6). This result suggests that genomes of species with large gene pools have developed more species-specific and lineage-specific genes compared to the core orthologs.

We investigated the levels of ortholog duplication per OG per genome or copy number of OG, *K*, which refers to a single OG of a genome comprising *K* different orthologs (Fig. 4b). Mean copy numbers of all four OG clusters were positively correlated with the number of genes (*r* = 0.71–0.81) (Supplementary Fig. S6). For example, copy numbers of the strict-core OGs in genomes with compact gene pools, such as the Bangiophyceae or Mamiellophyceae, are lower than those of their neighbor subgroups (Fig. 4a and c). These positive correlations between copy numbers of OGs and the number of genes may have resulted from genome duplications. For each OG, we estimated the number of gene duplication events (Supplementary Fig. S7). The strict-core (median of 197 duplications per OG) and the soft-core (median = 56) OGs have undergone more duplications compared to the shell (median = 13) and the cloud (median = 2) OGs. Overall, these results support that levels of duplication in orthologs are largely affected by genome duplication events, which increase with the depth of genealogy.

We investigated rates of missing and duplication in 7432 strict-core, soft-core, and shell OGs (Fig. 4d). Concordant with the large numbers of duplication events shown in the strict-core and soft-core OGs (Supplementary Fig. S7), an average of 95.76% of the strict-core OGs and 70.68% of the soft-core OGs existed as multi-copy across all genomes (Fig. 4b), whereas only 16.12% of the shell OGs existed as multi-copy. OG duplication rate was significantly correlated with BUSCO complete-duplicated rate (*r* = 0.56, *p*-value < 0.05), and OG missing rate was significantly correlated with BUSCO missing rate (*r* = 0.56, *p*-value < 0.05). Although we excluded genomes with BUSCO complete duplicated rates of 50% or higher during the representative genome selection (Fig. 2), 21 genomes showed high OG duplication rates with more than 50% of 7432 OGs (Fig. 4d). OG duplication rate was strongly correlated with the number of genes (*r* = 0.68, *p*-value < 0.05), whereas OG missing rate was not (*r* = − 0.33, *p*-value = 0.34) (Supplementary Fig. S6). Among nine genomes with OG missing rates of 70% or more, four genomes had showed unusually high missing rates compared to their taxonomic neighbors: *Digenea simplex* OPJA18 (78.44%), *Chromera velia* (83.41%), *Dunaliella* sp. M2 (74.70%), and *Hydrurus foetidus* (70.91%) (Fig. 4d). We note that high duplication/missing rates not agreeing with their phylogenetic neighbors could be artifacts of genome assembly and annotation, such as ploidy control and quality issues. Detailed duplication and missing of OGs are depicted in Supplementary Fig. S8.

In addition to the core orthologs, we also searched for lineage-specific OGs, referring to the OGs exclusively shared by the members of a subgroup. A total of eight subgroups with five or more representative genomes shared at least one lineage-specific OGs (Supplementary Fig. S9). Among eight subgroups, lineage-specific OGs shared by all members were only found in five taxonomic subgroups with a smaller number of members (n = 5–7). We could not detect any lineage-specific OG shared by all members in the subgroups containing larger numbers of members (n ≥ 12) (Supplementary Fig. S9). We summarized the top 20 most abundant PANTHER-linked Gene Ontology (GO) terms for the core OGs (strict-core and soft-core) and the lineage-specific OGs^29,30^ (Supplementary Fig. S10). The GO term abundance score of *N* indicates that the average of *N* copies of each gene was assigned to the GO term (Supplementary Fig. S10). GO terms enriched in the core OGs were assigned to the generic GO terms that are involved in the organismal fundamental^31^; for example, GO:0005737~cytoplasm, GO:0006412~translation, GO:0004672~protein kinase activity, GO:0016020~membrane, and GO:0016021~integral component of membrane (Supplementary Fig. S10). On the other hand, the most abundant GO terms for the lineage-specific OGs were differentiated by different subgroups while generally focused on specific GO terms, except for a few GO terms also enriched in the core OGs (Supplementary Fig. S10). This result indicates that the lineage-specific OGs are functionally distinguished between lineages and from the core algal OGs.

### The eukaryotic algal tree of life

**Figure 5:**
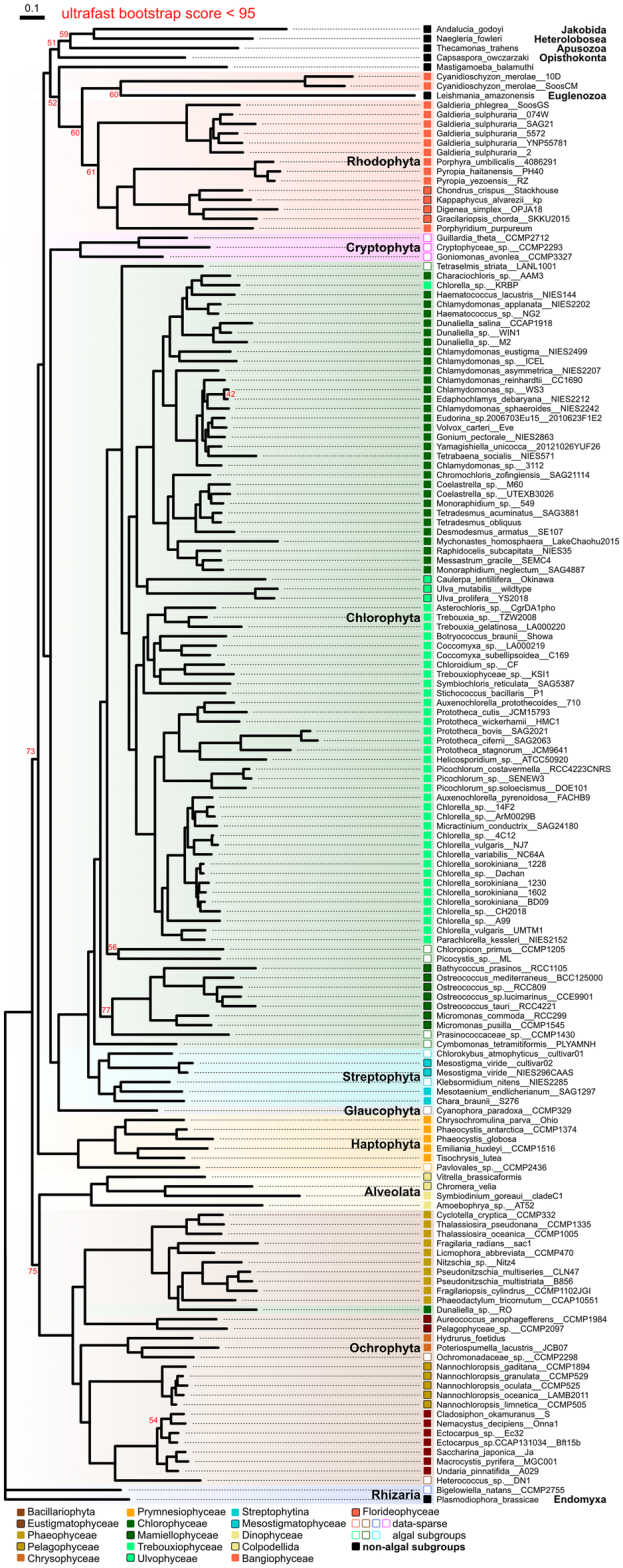
Maximum-likelihood tree reconstructed using 67 eukaryota_odb10 genes. Ultrafast bootstrap supports of 95% or more were omitted. Otherwise, bootstrap supports are denoted by nodes. Taxonomic groups are indicated by shaded rectangles. Taxonomic subgroups are indicated by a colored square.

Using 54 strict-core OGs identified in 149 representative algal genomes, we reconstructed a STAG species tree^32^ to maneuver high duplication rates in the strict-core OGs (Fig. 4e). The tree node support values for the STAG tree were not provided due to the small number of the strict-core OGs (< 100). In addition, we reconstructed a maximum-likelihood (ML) tree using 15,812 partitioned amino acid loci from 67 BUSCO eukaryota_odb10 genes (Fig. 5). For the ML tree, we added seven additional non-algal genomes from NCBI to properly locate eukaryotic algal clades. These genomes respectively represent the Endomyxa, Euglenozoa, Jakobida, Heterolobosea, Opisthokonta, Apusozoa, and Amoebozoa clades (Supplementary Table S4), which are clades previously used in a phylogenetic analysis of eukaryotic algal species^33^.

The ML tree displayed concordance between tree topologies and taxonomy at the subgroup-level (Fig. 4e). However, the STAG tree showed placements of genomes with high OG missing rates outside of their taxonomic subgroups: *Digenea simplex* OPJA18, *Chromera velia*, and *Hydrurus foetidus*. Interestingly, both STAG and ML trees supported misplacements of *Chlorella* sp. KRBP and *Dunaliella* sp. RO outside of their taxonomic groups (Figs. 4e and 5). Most of the genus-level clustering was consistent with the NCBI taxonomy database (Figs. 4e and 5). Although 40 of 149 representative algal genomes were taxonomically unclassified (see genome identifiers including “sp.” in the *Identifier* column of Supplementary Table S1), we could infer the taxonomic placements of these unclassified genomes based on their phylogenetic placements. For example, in the genus *Chlorella*, three of seven unclassified genomes were embedded with *Chlorella vulgaris* or *Chlorella sorokiniana* (Figs. 4e and 5). Still, the current data scarcity within the eukaryotic algal genome dataset compared to its taxonomic coverage makes it difficult to identify most unclassified algal species.

Unlike disrupted subgroup-level topologies in the STAG tree due to the highly missing genomes (Fig. 4e), group-level topologies of the ML species tree showed concurrence with the most recent eukaryotic algal phylogenies^33,34^ supported by strong ultrafast bootstrap values (Fig. 5). Other than the erroneous placement of the Rhodophyta group, clustering within Archaeplastida clade -Viridiplantae (Chlorophyta and Streptophyta), Rhodophyta, and Glaucophyta- and SAR clade -Stramenopiles (Ochrophyta), Alveolata, and Rhizaria- were concordant with previous reports. As suggested in previous studies^33,35,36^, the Cryptophyta group was placed with the Archaeplastida clade and the Haptophyta group was placed as a sister of the SAR clade (Fig. 5). However, the Rhodophyta group was placed outside of Archaeplastida clade with weak ultrafast bootstrap supports (Fig. 5). This result may result from the lack of eukaryota_odb10 genes in the Rhodophyta genomes related to the small gene pool of the Rhodophyta species: BUSCO complete rates of 37.3–76.9% (Fig. 2). Both phylogenetic methods used in this study have their limitations arising from the scarcity and quality of genomic resources. Nevertheless, using the largest set of eukaryotic algal genomes, our species trees provide us with a holistic view of algal phylogeny that correspond to the previous reports.

## Discussion

Our eukaryotic algal genome dataset revealed a major lack of structural annotations in currently available resources. Throughout this study, we (1) evaluated the cutting-edge annotation methods using high-quality eukaryotic algal genomes, (2) newly generated structural annotations for 135 genome assemblies without annotations, and (3) analyzed newly generated data to provide an expansive landscape of eukaryotic algal genomics (Fig. 1).

A total of 212 publicly available genome assemblies remained after our filtering steps, while more than 60% of them lacked structural annotations (Fig. 2). Among 77 annotated genomes, 21 of them were not annotated based on the extrinsic hints which are generally useful for predicting exon–intron boundaries (Fig. 2). Notably, the majority of the data-sparse algal clades were composed of unannotated genomes, which may have impeded high value of genomic resources in the data-sparse clades. For instance, in agreement with a previous study^34^, we observed low BUSCO genome complete rates (< 90%) across all algal taxa except for Chlorophyta, the sole eukaryotic algal phylum with a corresponding clade-specific OrthoDB set (Fig. 2). This result exemplifies that informational bias can affect the performance of contemporary genomics tools in the data-sparse clades. In a similar fashion, genome assemblies without structural annotations hinder researchers from accessing the full extent of biological knowledge. For these reasons, the inequality between the data-sparse clades and the others would have worsened. Thus, the low accessibility of structural annotations in the current eukaryotic algal genome dataset must be resolved.

By applying three annotation methods -AUGUSTUS-BUSCO, Braker-EP, and Braker-ES- to a set of high-quality genomes and OGSs (Fig. 3a), we showed that Braker-EP and Braker-ES methods performed well (Fig. 3b and c). Also, these two Braker-based methods were robust regardless of the continuity of genome assemblies (Supplementary Fig. S3). F1 scores of these structural annotations tended to decrease at more complex loci scope such as transcript-level (Fig. 3c), which has been already reported in previous benchmarks of ab initio and evidence-based gene annotations in non-model eukaryotes^15,37^. Although Braker-EP has been reported to be more accurate than Braker-ES with a model organism *D. melanogaster*^11^, it showed severely low F1s in our non-model eukaryotic genomes containing a small number of multi-exon genes (Fig. 3d). Braker-ES outperformed Braker-EP in these genomes, which indicates that Braker-ES can be an alternative when Braker-EP fails to perform an accurate spliced alignment of the protein hints to eukaryotic algal genomes. Since BUSCO complete rate represents the attainability of finding OrthoDB genes in the target genome^17^, we proposed a strategy to use the Braker-EP for genomes with high BUSCO complete rates (≥ 90%) and to use the Braker-ES method for genomes with low BUSCO complete rates (< 90%). Indeed, this strategy outperformed both Braker-ES and Braker-EP methods when used for a variety of non-model eukaryotic algal genomes (Supplementary Fig. S4). These evaluation results provide a benchmark for application of these cutting-edge annotation methods to a wide range of non-model eukaryotes, as well as its limitations. Using the mixed Braker strategy, we generated structural annotations for 135 unannotated genome assemblies, of which some are now to be the first structurally annotated genomes available for the species. Our approach is to liberate the algal research community from the constraints of extrinsic evidence data that is only accessible for a subset of algal species.

By utilizing newly generated gene sets, we built a large repertoire of eukaryotic algal genes and subsequently identified the algal core orthology. As our target species are distributed across a broad range of nine taxonomic groups (Fig. 4e), most of the OGs were shared by less than 15% of the genomes. The strict-core and soft-core clusters together, hereafter referred to as "core OGs", accounted for 556 OGs; this was a small number of OGs but still larger than the previously defined set (eukaryota_odb10, n = 255). These algal core orthologs were highly duplicated across all algal species (Fig. 4b and Supplementary Fig. S7). The core OGs were assigned to fundamental GO terms essential for organismal survival and also functionally distinct from the lineage-specific OGs (Supplementary Fig. S10). The high copy numbers and duplication rates, and fundamental functions of the core OGs across all species suggest that these gene duplications would have acted as an evolutionary buffer by providing room for lethal mutations in the core genes (Fig. 4c)^38^. Positive correlations between gene pool size and copy numbers of all OGs suggest that the genome duplication may have also contributed to a large number of duplicated orthologs (Supplementary Fig. S6). In a few representative genomes, we detected patterns of high rates of OG duplication and missing (Fig. 4d) that are inconsistent with their phylogenetic neighbor species, which requires further validation (Fig. 4e, and Supplementary Fig. S8). Duplication and missing of the OGs in these genomes were briefly suggested in the BUSCO genome assessment results (Fig. 2) but more notable in the OGs that have a better grasp of the target eukaryotic algal genes than a set of OrhotDB genes. Owing to the large number of eukaryotic algal genes used in this study, our algal orthology can provide guidance for evolutionary studies in eukaryotes, beyond the eukaryotic algae.

It is challenging to reconstruct the eukaryotic algal tree with a sampling bias present in the dataset. Additionally, relying on a single gene to reconstruct the phylogeny of such deep divergence can be erroneous due to confounding factors such as EGT/HGT, as shown in a previous phylogeny using the 26S proteasome regulatory complex subunit and SURF1^33,39^. Considering the ancient origin of the eukaryotic core orthologs, they would have been descended in genealogical manner rather than recently obtained via EGT/HGT. Therefore, we selected 149 representative genomes to minimize the sampling bias and selected multiple orthologs to reconstruct a multi-loci tree. However, all of the core OGs identified in this study exist as highly duplicated forms (Fig. 4b and c), which exponentially increases computation time of multiple sequence alignment for the classical phylogenetic tree reconstruction methods. To overcome the high duplication in the core orthologs, we reconstructed a STAG tree using the core OGs (Fig. 4e) and a multi-gene ML tree using the pre-defined set of OrthoDB genes (Fig. 5). Unlike the STAG tree, the ML tree showed clear phylogenetic-taxonomic congruence, particularly within-subgroup level topologies and the majority of group-level tree topologies (Fig. 5). It supports to the placements of different eukaryotic phyla of a broad range of algal and non-algal eukaryotes; for example, the placements of Cryptophyta and Haptophyta between the Archaeplastida and the SAR clade concur with the most recent reports of eukaryotic phylogeny^33,35,36^ (Fig. 5). Simultaneously, a robust species-level clustering points out taxonomic classifications of unclassified species, which is still one of the biggest challenges in the eukaryotic algal research. However, the Rhodophyta group was placed outside of the Archaeplastida in both trees (Figs. 4e and 5), possibly due to the lack of representation of Rhodophyta genes across the core ortholog and BUSCO eukaryota_odb10 datasets (Supplementary Fig. S1). Compared to the ML tree using BUSCO genes, the STAG tree using the core OGs showed more limitations. The misleading topologies of the core OG-based tree may have resulted from its sensitivity to the set of genome sequences used (Fig. 4e), which should be improved by securing more genomic resources for the data-sparse clades. In the same sense, misplacements of highly missing genomes (e.g., the Rhodophyta) in both ortholog-based phylogeny would be resolved by a eukaryotic ortholog set identified using phylogenetically balanced genomic data.

The results of this study will not only resolve user-end concerns regarding missing annotations and appropriate uses of the existing resources, but also lift the heavy computational burden of multiple genome annotations. The annotation methods used in this study are generalized to be used for any eukaryotic algal species, which makes it particularly useful for the data-sparse algal species. The evaluation results will provide a valuable guideline for non-model eukaryotic genome annotation. Last but not least, with an extensive set of eukaryotic algal genes generated in this study, we explored an undiscovered horizon of eukaryotic algal species that now includes the data-sparse algal clades. Thus, we believe that this study will play an essential role in the upcoming era of eukaryotic algal genomics.

## Materials and methods

### Genome data preparation and minimum quality check

We first retrieved genome sequences of “eukaryotic algae” from public genome databases including the NCBI GenBank assembly database^40^ and the JGI database^41^. We used the Entrez tool^42^ implemented in the Biopython package^43^ to retrieve data from the NCBI GenBank assembly database (accessed July 30, 2020). Genome assemblies from other public genome databases were manually obtained. Genome annotations and protein sequences corresponding to the genome assemblies were also retrieved. Details of the dataset is summarized in Supplementary Tables S1 and S2.

We excluded genome assemblies that did not pass our minimum quality check criteria. We excluded partial genomes or genomes from unreliable sources such as metagenome, environmental, and single-cell sources (Supplementary Table S2). Next, we calculated the median length of eukaryotic algal genes from 17 highly continuous NCBI genome assemblies with contig N50 values of 100 Kb or more. Accordingly, genome assemblies with contig N50 values less than the median gene length were excluded^21^ (Supplementary Table S2). In addition, we excluded genome annotations with (1) anomalies in core annotation features and (2) discordances between genome assembly, structural annotation, and protein sequences (Supplementary Table S1).

### BUSCO assessments for genome assemblies and annotations

We performed BUSCO analysis v4.0.6^17^ to assess the quality of the genome assembly and annotation for downstream analyses. We generated BUSCO genome mode (-m genome) using OrthoDB v10 with auto lineage selection enabled (--auto-lineage-euk)^27^. We excluded genome assemblies with BUSCO missing rates of 75% or more (Supplementary Table S2). For protein sequences extracted based on structural annotations, we additionally performed the BUSCO protein assessment (-m protein) with the BUSCO lineages automatically selected in BUSCO genome mode. Genomes with annotations that failed any quality check were further re-annotated. All results were summarized using the ggplot2 package^44^ of R^45^.

### Gene prediction methods

We selected three gene prediction methods that can be universally used for any algal species without a requirement of species-specific data. To train a species-specific gene model for each genome, we used AUGUSTUS v3.3.3^9^ and Braker v2.1.6^11^ (Fig. 1).

1. *AUGUSTUS-BUSCO*: We performed BUSCO genome mode with auto lineage selection (–auto-lineage-euk). With automatically selected OrthoDB lineage, the BUSCO pipeline trained AUGUSTUS gene model using BUSCO complete genes. Then, we performed ab initio gene prediction using AUGUSTUS with the BUSCO-based gene model (--species = BUSCO-based gene model). The following parameters were used according to the authors' suggestion: --strand = both, --genemodel = complete, --protein = on, --alternatives-from-sampling = true, --minexonintronprob = 0.1, --minmeanexonintronprob = 0.4, --gff3 = on. Protein sequences of the predicted genes were then extracted from genome sequences using getAnnoFasta.pl of the AUGUSTUS package.
2. *Braker-ES*: We performed Braker2 gene prediction with ab initio gene finding algorithm GeneMark-ES (--esmode)^14^. We lowered the minimum contig size to 10 Kb (--min_contig = 10000) and enabled softmasked region detection for softmasked genomes (--softmasking).
3. *Braker-EP*: We performed Braker2 gene prediction with extrinsic evidence of protein sequences of any evolutionary distance using GeneMark-EP/EP+, ProtHint, Spaln2, and DIAMOND^12,36,46^. To generate extrinsic protein hint files, we first extracted clade-specific orthologous gene sets of the following algal clades from the OrthoDB v10.0 database^27^: Eukaryota, Chlorophyta, Chlorophyceae, Rhodophyta, and Bacillariophyta. For each clade, we collected all protein sequences in OrthoDB v10.0 OGs that are shared by 80% or more of species of the clade. We then performed Braker2 gene prediction using the protein hint (--epmode, --prot_seq) with the same parameters used in Braker-ES (--min_contig = 10000, --softmasking). Based on the taxonomic classification of each genome, we used protein hints of the most specific clade.

### Evaluation of the gene prediction methods in eukaryotic algae

Based on the results of BUSCO genome and protein assessments, we selected 45 “qualified” genomes and OGSs that (1) showed BUSCO missing rates less than 25% in both genome and protein modes and (2) were generated using any types of extrinsic evidence (e.g., RNA-seq). For these “qualified” genome assemblies, we evaluated different gene prediction methods against their OGSs and three default AUGUSTUS gene models (AUGUSTUS-default): AUGUSTUS-chlamydomonas, a default gene model trained on *Chlamydomonas reinhardtii* gene set; AUGUSTUS-galdieria, a default gene model trained on *Galdieria sulphuraria* gene set; AUGUSTUS-tetrahymena, a default gene model trained on *Tetrahymena thermophila* gene set.

We compared BUSCO protein mode on OGS, AUGUSTUS-default gene model, and gene models generated from all three gene prediction methods. In addition, GffCompare v0.11.2^23^ was used to calculate the accuracy metrics of the predicted gene sets against the OGS with the following option: -R (ignore non-overlapping reference transcripts) and -Q (ignore non-overlapping query transcripts). At each loci level, sensitivities (or recall; the ratio of true positive matches to the OGS) and precisions (the ratio of true positive matches to newly predicted genes) were calculated. F1 was calculated as the harmonic mean of sensitivity and precision. Pearson’s correlation analyses and Wilcoxon rank-sum test^47^ were performed in R. All results were summarized using the ggplot2 package of R.

### Representative genome selection

To prevent phylogenetic redundancy, we selected representative genomes using BUSCO and MASH^26^. We first performed MASH pair-wise analyses (mash triangle) using all genomes with 10000 hash sampling (-s 10000). Based on the MASH distance, we clustered genomes with more than 96% similarity. After excluding highly missing or highly duplicated genomes (BUSCO missing rate > 75% or BUSCO complete duplicated rate > 50%), we selected one representative genome with the best BUSCO complete rates within each cluster.

### Orthology identification and functional annotation

With 149 representative genomes, we identified the orthology of genes and reconstructed the species tree using Orthofinder v2.4.0^48^. We chose the sequence similarity search tool DIAMOND BLAST^49^ to identify OGs, which refers to a group of genes descended from a single common ancestral gene. OGs were grouped into four clusters based on the number of genomes sharing the OG: “strict-core” (100% of genomes), “soft-core” (≥ 95%), “shell” (≥ 15%), and “cloud” OGs (< 15%).

To collect lineage-specific OGs, we selected taxonomic subgroups with five or more members. To outline the functional overview of the core OGs and lineage-specific OGs, we performed functional annotation of the OGs shared by all members of the subgroup. We used InterproScan v5.46^50^ with the PANTHER v1.4.1 database (-appl PANTHER)^29^ and PANTHER-linked-GO terms (-iprlookup, -goterms)^30^. Correlation analyses between statistics were performed using Pearson’s method in R. All results were summarized using the ggplot2 package of R.

### Phylogenomic tree reconstruction

Seven additional genome assemblies of non-algal species were retrieved from the NCBI database for the phylogenomic analysis. We performed BUSCO genome mode using eukaryota_odb10 on all genomes, then extracted eukaryota_odb10 genes shared by more than 80% of the genomes. With these protein sequences, we performed multiple sequence alignment using MAFFT v7.471^51^ and removed loci with missing rates of > 20%. We merged all sequence alignments into a concatenated alignment partitioned by each gene. With the partitioned alignment, we used ModelFinder to find the best-fit evolutionary model that corresponds to the partition scheme (-m MFP + MERGE)^52^. Using the best-fit model, we reconstructed the maximum-likelihood tree using IQ-TREE v2.0.3^53^ with empirical amino acid frequency (-mfreq F) and 1000 replicates of ultrafast bootstrap^54^.

In parallel, a STAG tree was reconstructed using the strict-core OGs found during the Orthofinder analysis. As there was no single-copy OGs identified with more than 50% genomes involved, ML tree reconstruction was not performed. Instead, we used DendroBLAST^41^ to infer individual gene trees that were further merged using the STAG method^32^, which can infer species trees based on individual gene trees while fully employing highly duplicated OG information. The minimum requirement of the number of the strict-core OGs was not satisfied (< 100), and the tree node support values were not generated. All phylogenomic trees were visualized with EvolView v3^55^.

## Supplementary Information

Supplementary Information.

Supplementary Table S1.

Supplementary Table S2.

Supplementary Legends.

## Data Availability

Access to the publicly available genome sequences (FASTA), genome annotations (GFF), and protein sequences (FASTA) used in this study are described in Supplementary Table S1. Annotations (GFF) and corresponding protein sequences (FASTA) newly generated in this study are available in *Mendeley Data* 10.17632/b32nw6rrfh.1.
