# Supplementary material for: Addressing the pervasive scarcity of structural annotation in eukaryotic algae
Source: Sci Rep. 2023 Jan 30;13:1687. doi: 10.1038/s41598-023-27881-0 (PMC9886943; doi:10.1038/s41598-023-27881-0)
Supplement: Supplementary file 1 — Supplementary Information. [file 41598_2023_27881_MOESM1_ESM.pdf]

Supplementary Information

Addressing the pervasive scarcity of structural annotation in eukaryotic algae

Taehyung Kwon[1], Erik R. Hanschen[1], Blake T. Hovde[1]*

[1] Genomics and Bioanalytics, Bioscience Division, Los Alamos National Laboratory, New Mexico, United States

* Corresponding author

E-mail address: hovdebt@lanl.gov

Supplementary Figures

Figure S1. The result of BUSCO genome mode assessment using eukaryota_odb10.

Figure S2. The comparison of BUSCO protein mode missing rates between the official gene set and newly predicted gene sets.

Figure S3. Correlation analyses of F1 scores of Braker-EP and Braker-ES.

Figure S4. Average F1 scores of Braker-EP/Braker-ES mixed strategy.

Figure S5. Histogram of the number of orthogroups depicted by the number of species sharing each orthogroup.

Figure S6. Correlation analyses between various orthology statistics.

Figure S7. The number of duplication events with 50% support for each cluster of orthogroup.

Figure S8. Heatmap of duplication and missing orthogroups in the strict-core, soft-core, and shell orthogroup clusters.

Figure S9. The number of lineage-specific orthogroups.

Figure S10. The top 20 most abundant Gene Ontology terms found in the core orthogroups and the lineage-specific orthogroups.

Supplementary Tables

Table S3. Summary of the OrthoFinder analysis.

Table S4. List of non-algal genomes used in the phylogenetic analysis.

Figure S1. The result of BUSCO genome mode assessment using eukaryota_odb10.

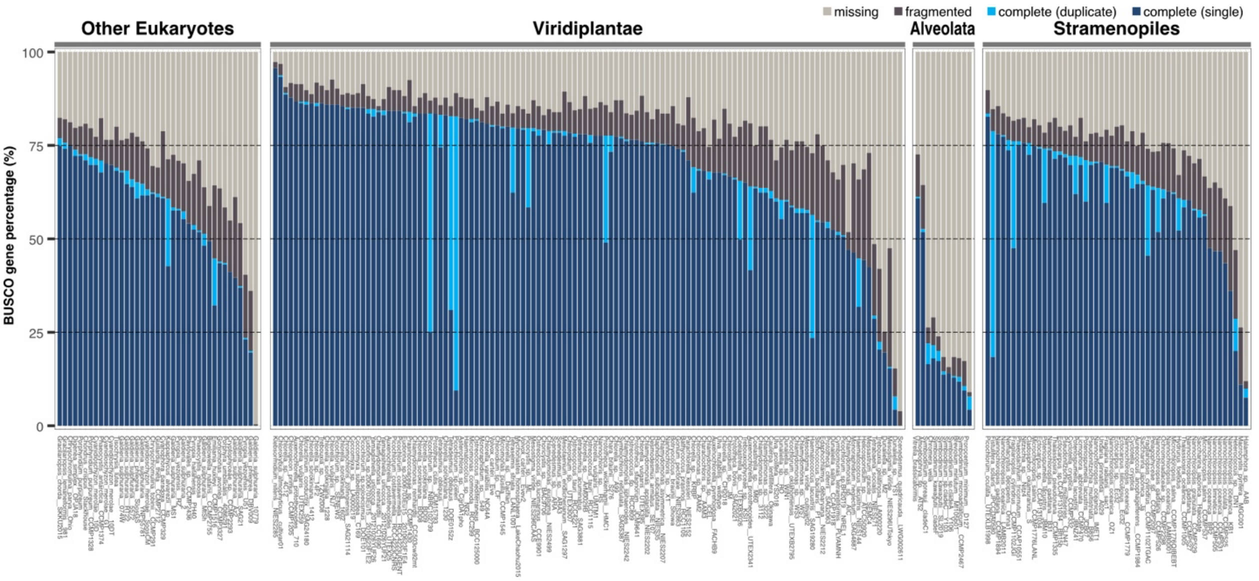

Figure S2. The comparison of BUSCO protein mode missing rates between the official gene set and newly predicted gene sets. Positive value indicates that the newly predicted gene set outperformed the official gene set.

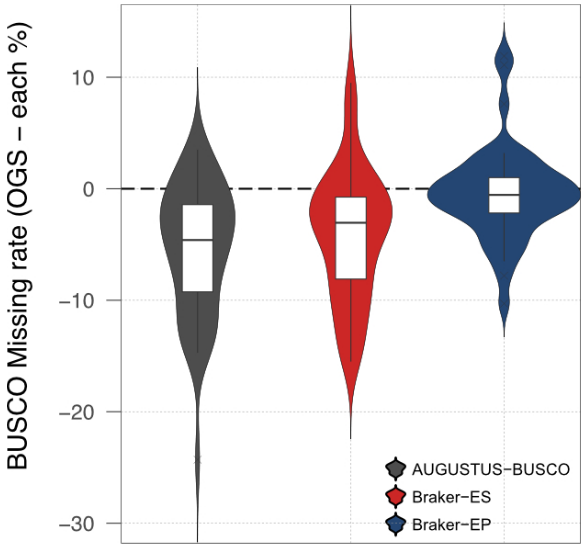

Figure S3. Correlation analyses of F1 scores of Braker-EP and Braker-ES. At base, exon, and transcript loci level, F1 score of each genome is illustrated by genome size, contig N50, BUSCO complete rate, respectively. Pearson's correlation coefficients are highlighted in blue if statistically significant (*p*-value < 0.05).

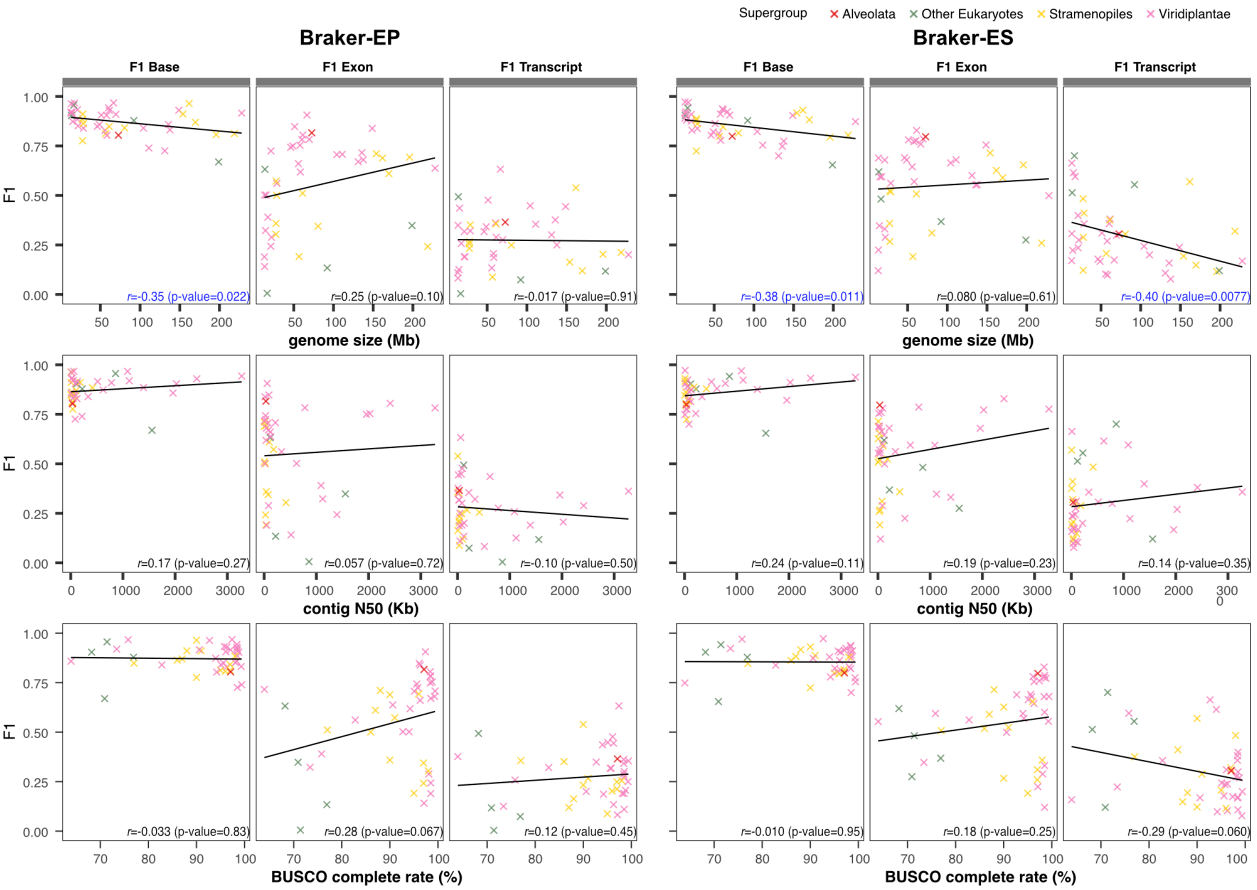

Figure S4. Average F1 scores of Braker-EP/Braker-ES mixed strategy. Braker-EP was used for genomes with BUSCO complete rate of the cutoff or higher. Braker-ES was used for genomes with BUSCO complete rate lower than the cutoff. Error bars indicate upper and lower standard deviation ranges, respectively. Blue dots indicate the maximum F1 score at each loci level. Red dashed line indicates the cutoff used for the downstream analyses in this study.

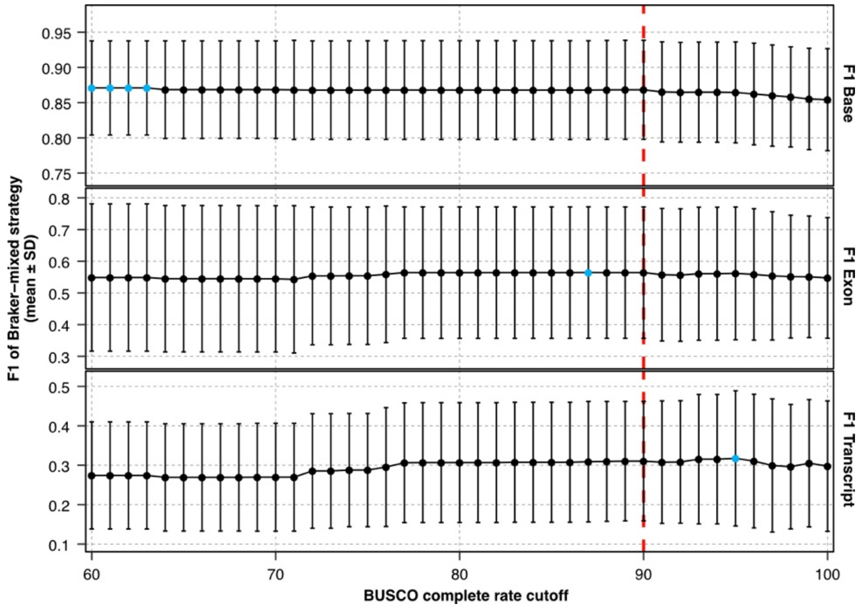

Figure S5. Histogram of the number of orthogroups depicted by the number of species sharing each orthogroup.

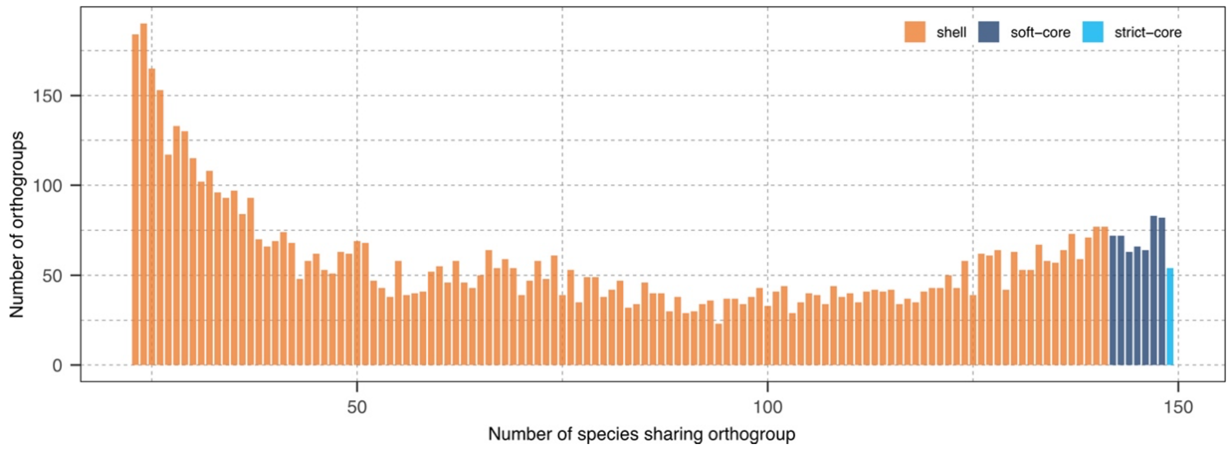

Figure S6. Correlation analyses between various orthology statistics. The lower diagonal displays Pearson's *r* values. The upper diagonal denotes *p*-values of each Pearson's correlation test.

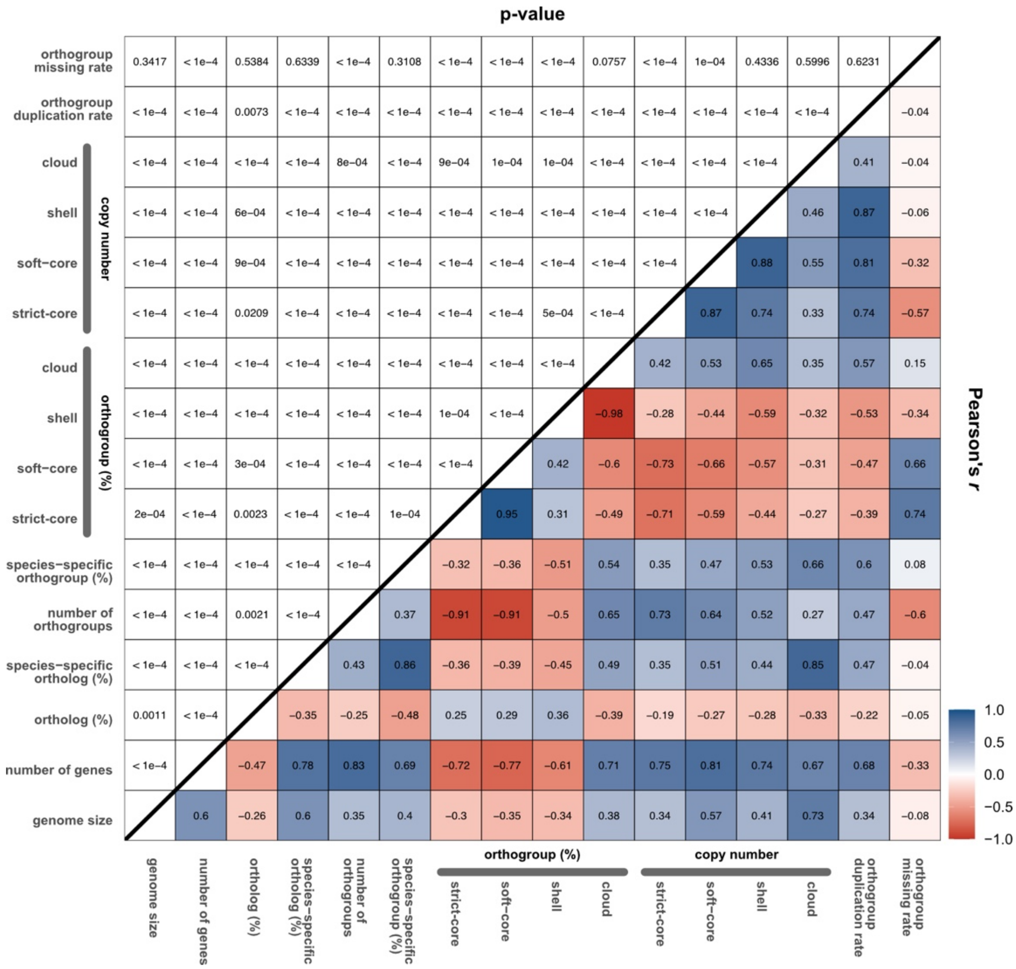

Figure S7. The number of duplication events with 50% support for each cluster of orthogroup. A duplication event with 50% support indicates that both copies of the duplicated gene are retained by at least 50% of the descendants.

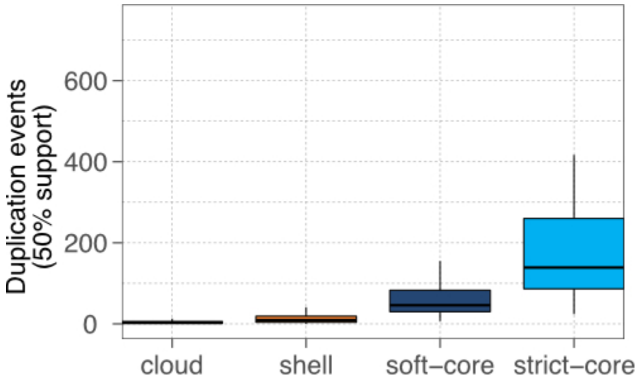

Figure S8. Heatmap of duplication and missing orthogroups in the strict-core, soft-core, and shell orthogroup clusters.

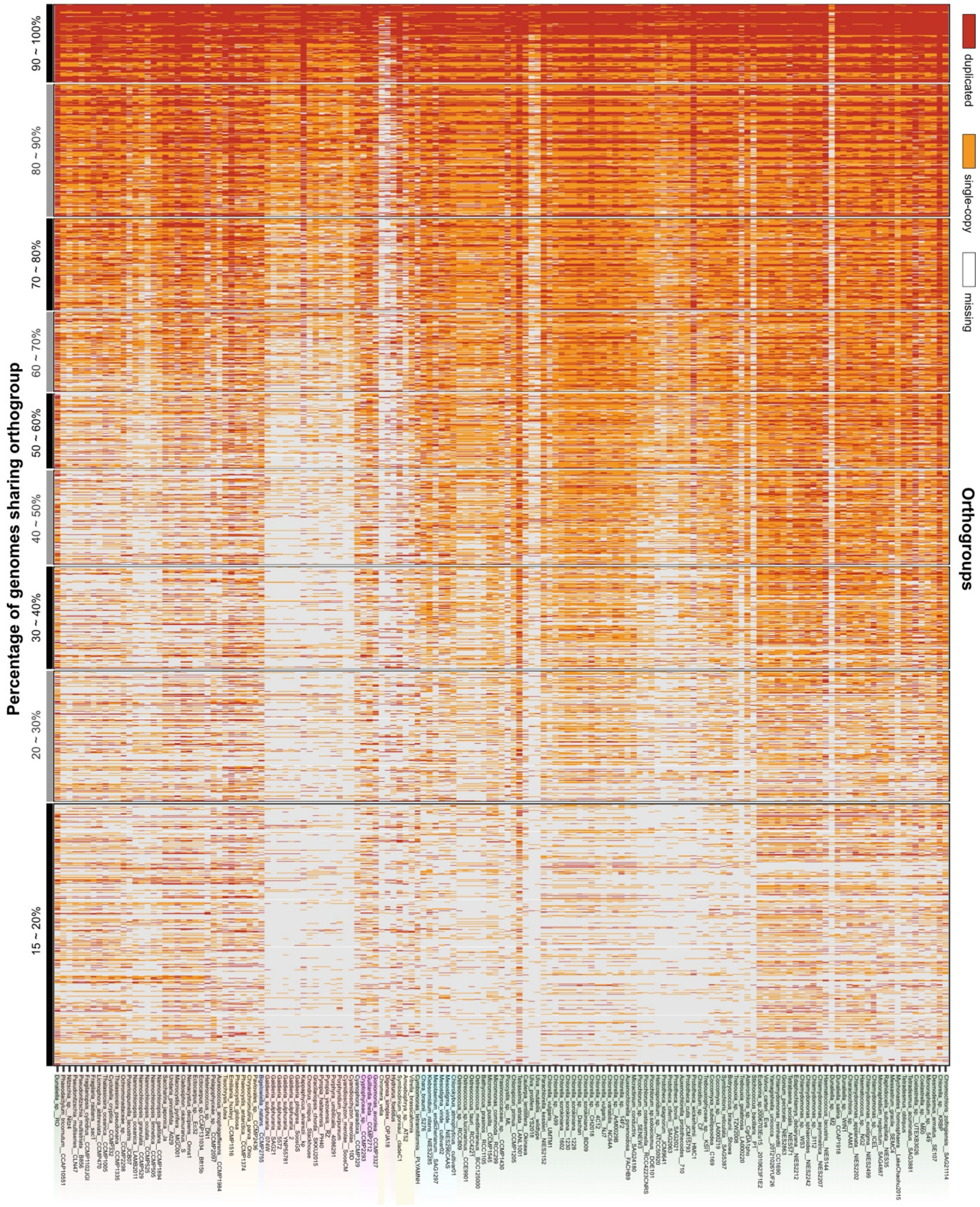

Figure S9. The number of lineage-specific orthogroups. Bars indicate the number of orthogroups exclusively found in more than 50% of the members of the subgroups composed of five or more genomes.

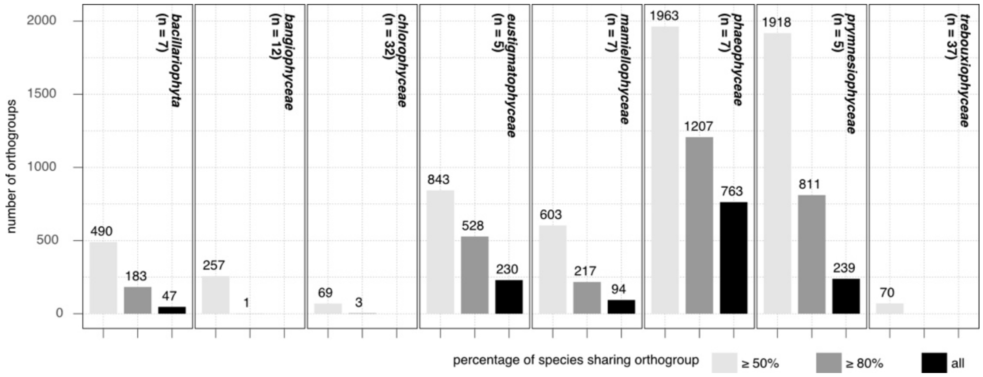

Figure S10. The top 20 most abundant Gene Ontology terms found in the core orthogroups and the lineage-specific orthogroups. The GO term abundance of *N* indicates that the average of *N* copies of each gene in a orthogroup were assigned to the GO term.

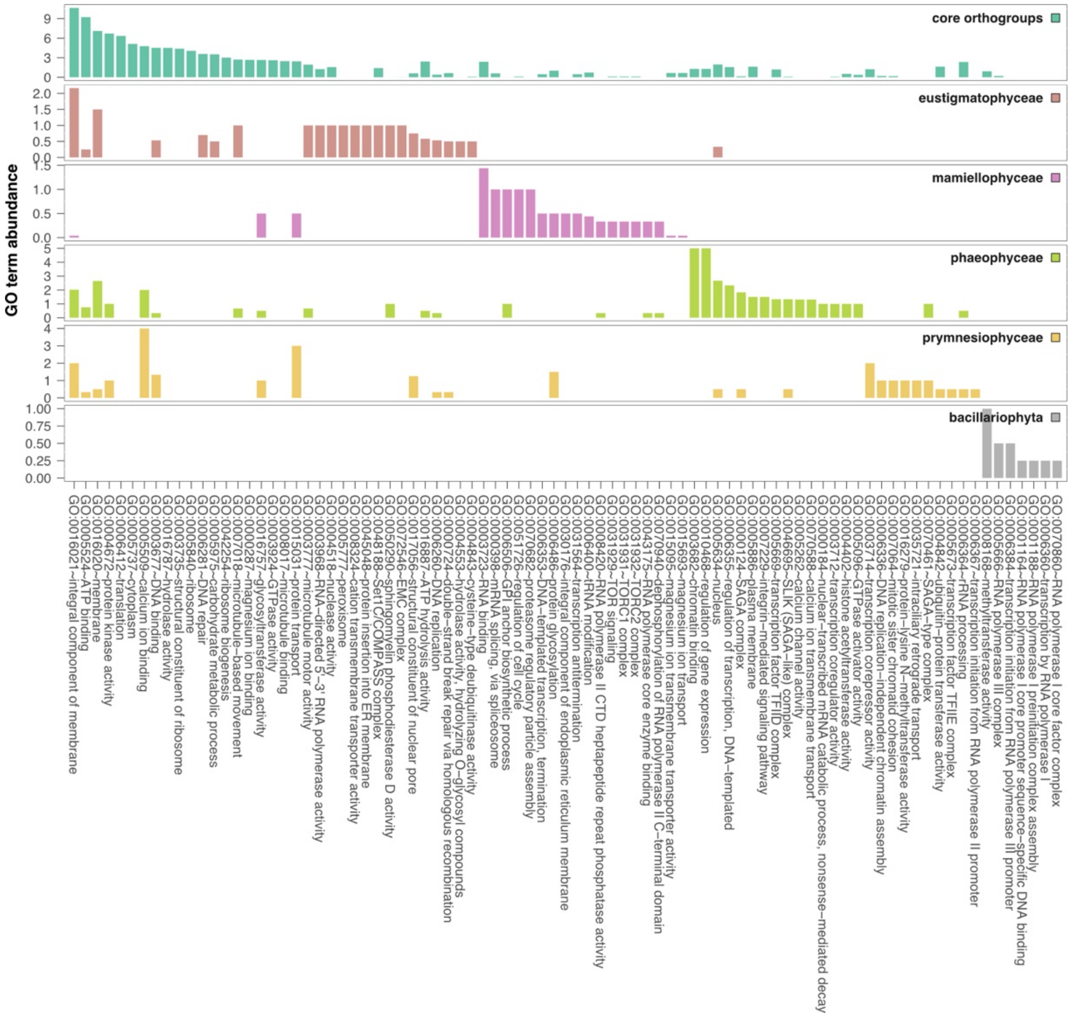

Table S3. Summary of the OrthoFinder analysis.

Statistics	
**Total number of genes**	2288060
**Total number of orthogroups**	116433
**The number of Strict-core orthogroup**	54
**The number of Soft-core orthogroup**	502
**The number of Shell orthogroup**	6876
**The number of Cloud orthogroup**	109001
**The number of Species-specific orthogroups**	46824
**The number of genes in orthogroups**	2061744
**The number of genes in species-specific orthogroups**	242076

Table S4. List of non-algal genomes used in the phylogenetic analysis.

Identifier	NCBI Accession	Group	Subgroup	Species
Plasmodiophora_brassicae	GCA_003833335.1	Rhizaria	Endomyxa	*Plasmodiophora brassicae*
Leishmania_amazonensis	GCA_005317125.1	Discoba	Euglenozoa	*Leishmania amazonensis*
Andalucia_godoyi	GCA_009859145.1	Discoba	Jakobida	*Andalucia godoyi*
Naegleria_fowleri	GCA_008403515.1	Discoba	Heterolobosea	*Naegleria fowleri*
Capsaspora_owczarzaki	GCA_000151315.2	Opisthokonta	Opisthokonta	*Capsaspora owczarzaki*
Thecamonas_trahens	GCA_000142905.1	Apusozoa	Apusozoa	*Thecamonas trahens*
Mastigamoeba_balamuthi	GCA_902651635.1	Amoebozoa	Amoebozoa	*Mastigamoeba balamuthi*
