# Supplementary material for: Addressing the pervasive scarcity of structural annotation in eukaryotic algae
Source: Sci Rep. 2023 Jan 30;13:1687. doi: 10.1038/s41598-023-27881-0 (PMC9886943; doi:10.1038/s41598-023-27881-0)
Supplement: Supplementary file 4 — Supplementary Legends. [file 41598_2023_27881_MOESM4_ESM.docx]

**Supplementary Table S1 (.xlsx)**

Table S1. Summary of eukaryotic algal genome assemblies used in this study.

**Supplementary Table S2 (.xlsx)**

Table S2. Summary of eukaryotic algal genome assemblies excluded from this study.
